# Supplementary material for: DMRforPairs: identifying Differentially Methylated Regions between unique samples using array based methylation profiles
Source: BMC Bioinformatics. 2014 May 15;15:141. doi: 10.1186/1471-2105-15-141 (PMC4046028; doi:10.1186/1471-2105-15-141)
Supplement: Additional file 2 — DMRforPairs output for the comparison of A431-MCF7 and NA17018-NA17105. Please start from the HTML files in each folder. Available via the BMC Bioinformatics website. [file 1471-2105-15-141-S2.zip › 1394847754114233_MOESM2_ESM/A431_MCF7/figures/34126.pdf]

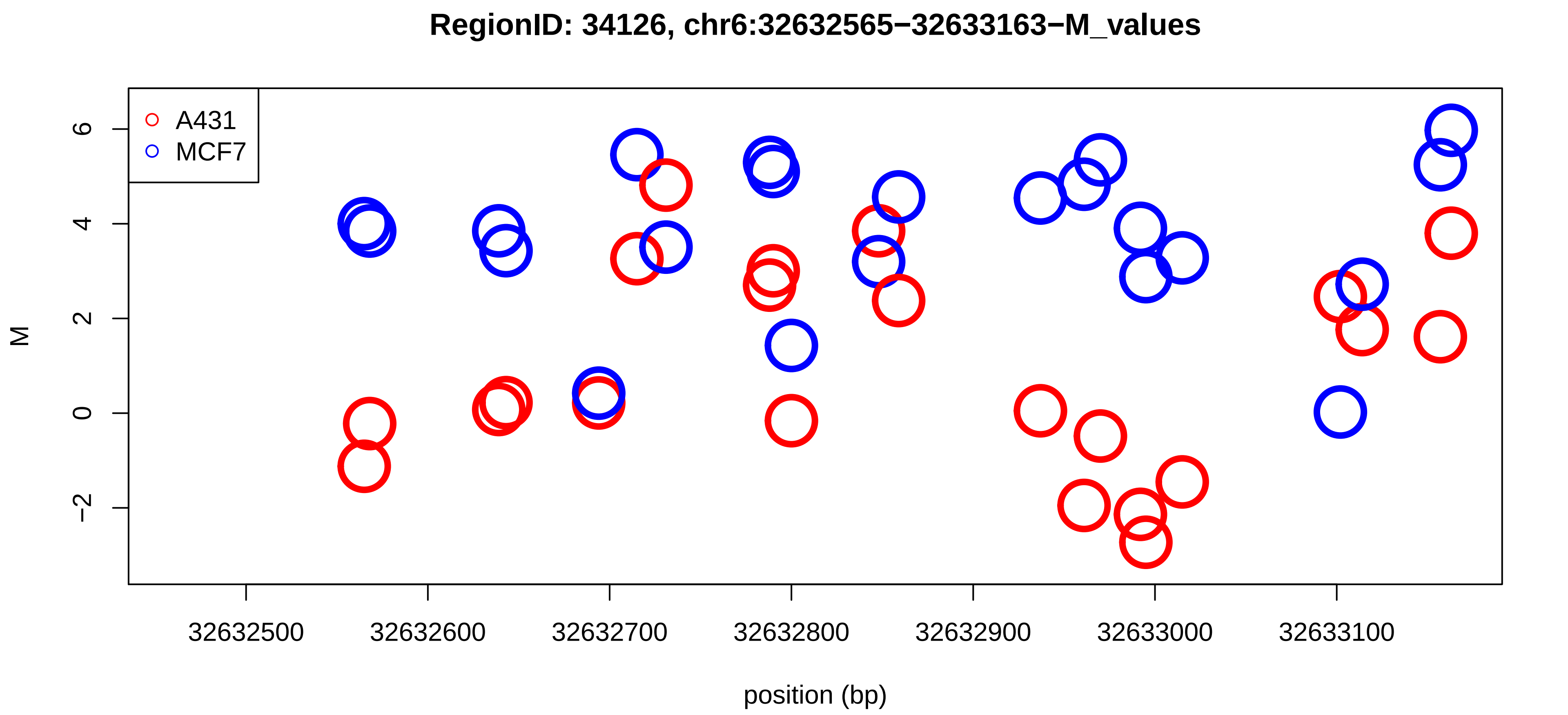
RegionID: 34126, chr6:32632565−32633163−M_values
A431
MCF7
M
6
4
2
0
−2
32632500
32632600
32632700
32632800
32632900
32633000
32633100
position (bp)

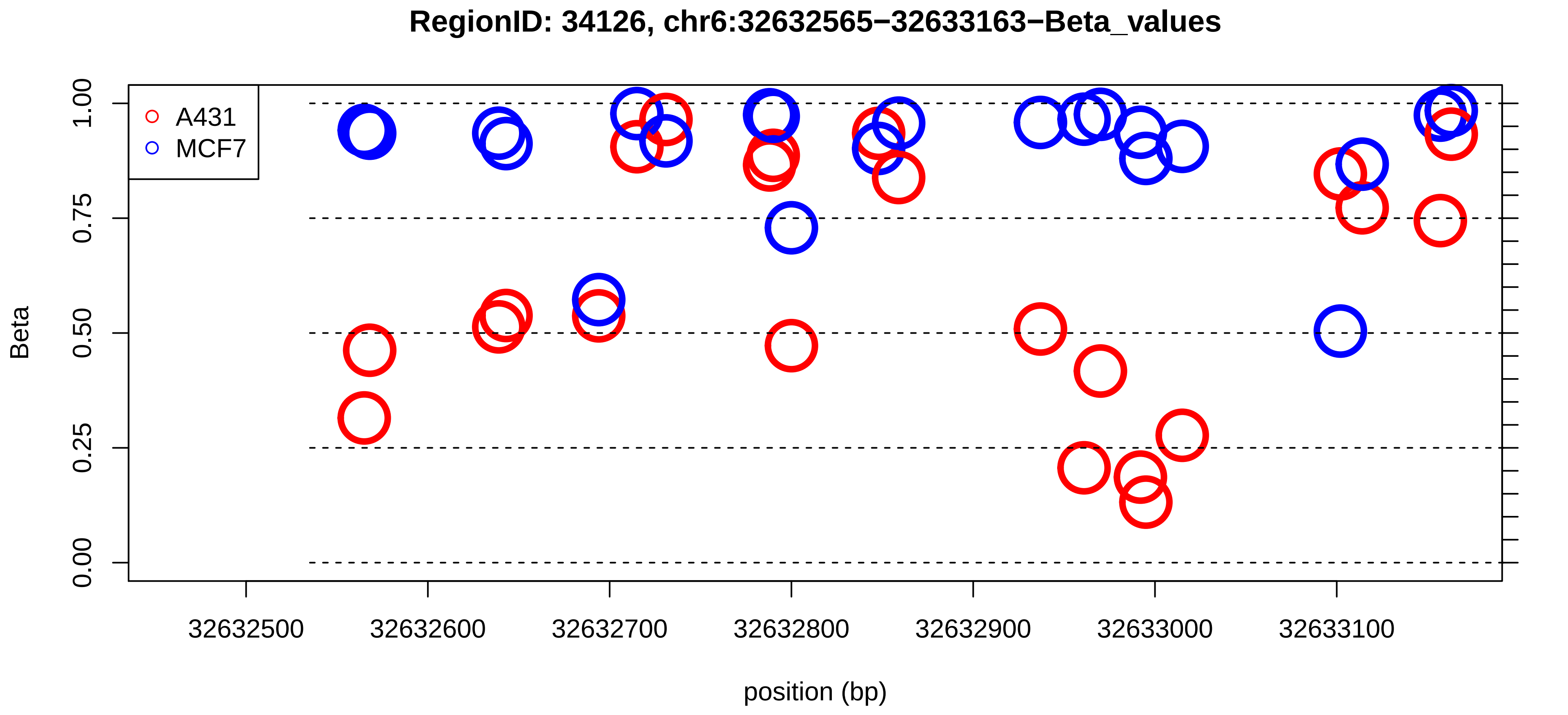
RegionID: 34126, chr6:32632565−32633163−Beta_values
A431
MCF7
Beta
1.00
0.75
0.50
0.25
0.00
32632500
32632600
32632700
32632800
32632900
32633000
32633100
position (bp)

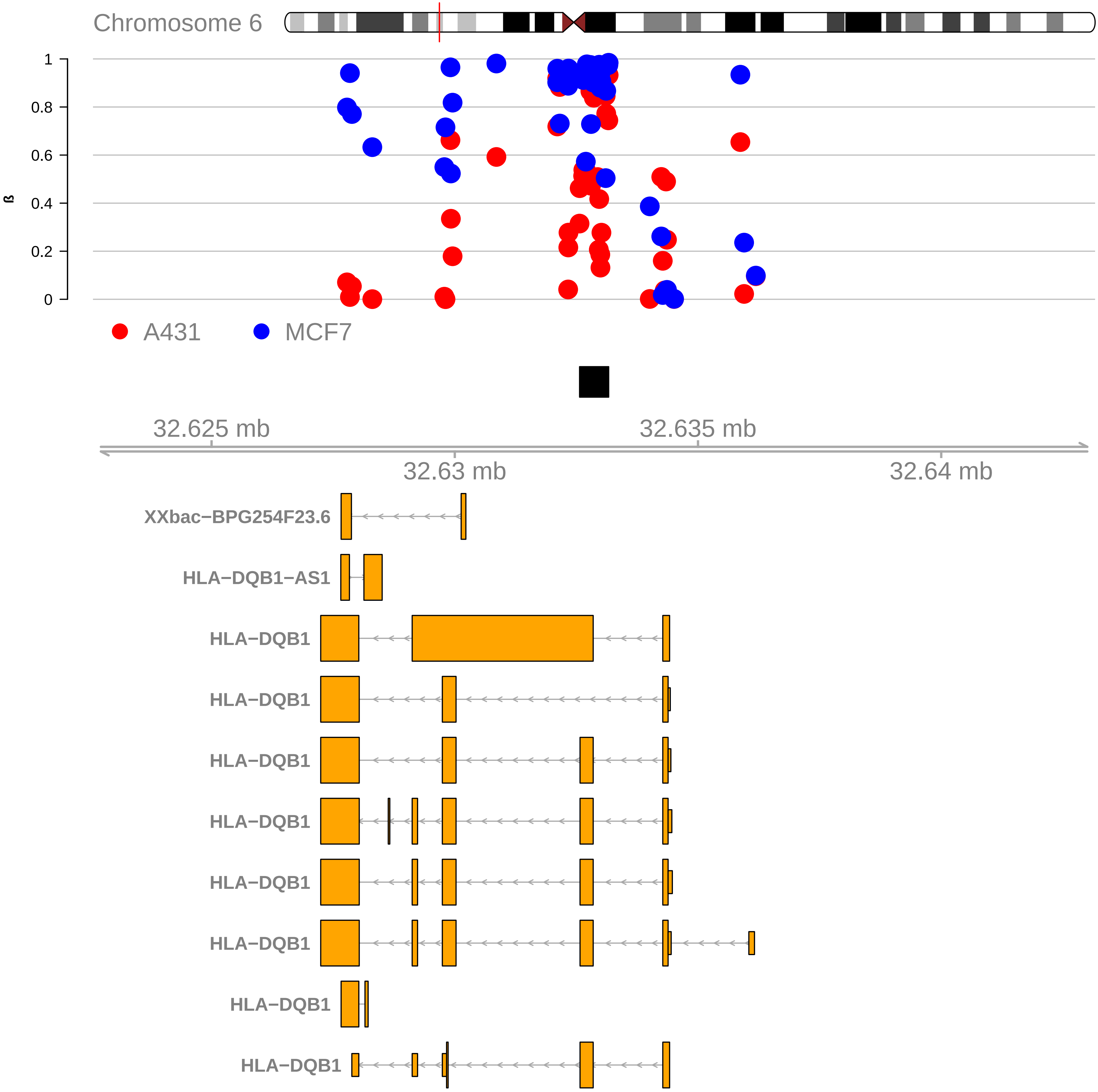
Chromosome 6
1
0.8
0.6
0.4
0.2
0
β
A431
MCF7
32.625 mb
32.635 mb
32.63 mb
32.64 mb
XXbac−BPG254F23.6
HLA−DQB1−AS1
HLA−DQB1
HLA−DQB1
HLA−DQB1
HLA−DQB1
HLA−DQB1
HLA−DQB1
HLA−DQB1
HLA−DQB1
